# Genetic variation in PSCA gene and bladder cancer susceptibility in North Indian population

**DOI:** 10.1186/1755-8166-7-S1-P15

**Published:** 2014-01-21

**Authors:** Praveen K Jaiswal, Vibha Singh, Rakesh Kapoor, Rama D Mittal

**Affiliations:** 1 Department of Urology and Renal Transplantation, Sanjay Gandhi Post Graduate Institute of Medical Science, Raebareli Road, Lucknow 226014, Uttar Pradesh, India

## Background

Bladder cancer (BC) is a significant health problem worldwide. Prostate stem cell antigen (PSCA) gene has been reported earlier in Genome Wide Association Study (GWAS) for BC risk. It is highly expressed in bladder cancer and considered to be involved in the cell proliferation inhibition and/or cell-death induction activity. It has been assumed as a useful marker for diagnosis and progression of bladder cancer.

## Materials and methods

We genotyped PSCA rs2978974G/A and PSCA rs2294008C/T gene by Real Time Taqman® probes to evaluate the risk of bladder cancer (BC) in histologically confirmed 225 BC patients and 240 healthy controls (age and gender matched) from North Indians in a hospital based case control study. Further statistical analysis for association studies was done by SPSSver16.0.

## Results

Significant increased BC risk was observed to be associated with heterozygous CT genotype of PSCA rs2294008C/T having 1.86 folds risk (p=0.004;OR=1.86). The variant allele T was also significantly associated with BC risk (p=0.027;OR=1.38) for PSCA rs2294008C/T. In case of PSCA rs2978974G/A, no significant association was observed with BC at genotypic level. Smoking significantly modulated the BC risk in patients with heterozygous CT genotype (p=0.025) for PSCA rs2294008C/T gene polymorphism. A significant BC risk was observed when risk was evaluated with tumor-grade-stage level for PSCA rs2294008C/T with heterozygous CT genotype (p=0.045;OR=1.02). Furthermore, BC patients receiving BCG treatment showed no significant association with any genotype of PSCA. Bioinformatics analysis (in-silico analysis) showed no significant association with BC risk.

## Conclusions

Our study has unveiled a complex intervention of PSCA rs2294008C/T conferring a higher risk of BC risk among North Indian population. Further studies in large sample size and different ethnic group are needed for validation.

